# Single-Mode Capability Enhancement of Curved Sapphire Fiber Utilizing High-Order Mode Suppression Characteristics Applied at High Temperature

**DOI:** 10.3390/mi17060748

**Published:** 2026-06-21

**Authors:** Fan He, Chengkuo Lee, Xiaojin Zhang, Jiamin Chen, Yongqiu Zheng, Chenyang Xue

**Affiliations:** 1 State key Laboratory of Extreme Environment Optoelectronic Dynamic Measurement Technology and Instrument, North University of China, Taiyuan 030051, China; sz202306102@st.nuc.edu.cn (F.H.); 13834383024@163.com (X.Z.); zhengyongqiu@nuc.edu.cn (Y.Z.); xuechenyang@nuc.edu.cn (C.X.); 2 Center for Intelligent Sensors and MEMSECE Department, National University of Singapore, Singapore 117576, Singapore; elelc@nus.edu.sg

**Keywords:** curved sapphire fiber, single-mode capability, high-order mode curved losses, high temperature

## Abstract

In this paper, a comprehensive investigation into the single-mode capability of curved sapphire fiber is performed, ranging from theoretical simulation to experimental verification. The equivalent refractive index theoretical model for curved sapphire fiber is proposed based on stress–optic effects and the conformal mapping technique. According to the finite element method, when the radius of curvature is 0.02 m, the curved losses’ difference between high-order modes and the fundamental mode is as high as five orders of magnitude, demonstrating the best single-mode potential. In addition, the curving experiments of sapphire fiber and sapphire fiber Bragg grating are completed. The transmission spectrum of the curved sapphire fiber with a curving radius of 0.02 m is the closest to that of the single-mode fiber. As for curved sapphire fiber Bragg grating (CSFBG), the 3 dB bandwidth of reflection spectrum with the same radius of curvature is also the smallest, with a value of 3.7 nm. Furthermore, the temperature performance of the proposed CSFBG is measured from 22 °C to 1600 °C. The sensitivity is 37.88 pm/°C (@1600 °C), and the measurement accuracy is ±2.98 °C. This study provides theoretical support for single-mode signal transmission of curved sapphire fibers and facilitates high-precision sensing applications under extreme high-temperature conditions.

## 1. Introduction

In advanced national defense industry fields such as the combustion chambers of aero-engines and the cores of nuclear reactors, there are working environments with temperatures exceeding 1800 °C [1,2,3,4]. Under these extreme high temperature conditions, in situ sensing technology can directly measure temperature and stress distribution [5,6], which is critical for assessing the structural reliability and safety of combustion chambers. Precise in situ sensing technology provides key data for reactor safety monitoring, service life assessment, structural optimization, and early fault warning. Due to the inherent material properties of traditional silica fibers, they cannot maintain stable performance at temperatures above 1100 °C. The sapphire fiber with a melting point exceeding 2053 °C has emerged as the ideal material for ultra-high-temperature optical fiber sensing applications. However, the core diameter of the sapphire fiber prepared by the Laser Heating Pedestal Growth (LHPG) technique is relatively large, and there is no suitable thermally matched cladding material. This results in multimode propagation characteristics of the sapphire fiber [7]. The number of modes in sapphire fiber with a diameter of 60 μm can be as high as 15,000. The contrast of the interference fringes and the measurement accuracy of the sapphire fiber sensor will be reduced by the excited higher-order modes (HOMs). For instance, the sapphire fiber Bragg grating (SFBG) exhibits large-bandwidth asymmetric reflection spectra (with a bandwidth of 6 nm), far exceeding that of the single-mode fiber Bragg grating (SMF-FBG) (with a bandwidth of 0.7 nm) [8,9]. Accordingly, the temperature measurement accuracy of SFBGs below 700 °C is ±10 °C [10], while that of SMF-FBGs is ±1.8 °C. Therefore, technology for the development of single-mode sapphire fibers is crucial for improving sensing accuracy in high-temperature environments.

At present, some research has been conducted on techniques for the development of single-mode sapphire fibers [11,12,13,14,15,16,17,18,19]. Initially, researchers suppressed HOMs by reducing the core diameter. C. Hill and X. Tao applied high-temperature wet acid etching and the state-of-the-art LHPG system to produce sapphire fibers with minimum diameters of 0.8 μm and 16 μm [20,21,22,23], respectively. The prepared sapphire fibers with extremely thin diameters are difficult to use in actual harsh environments. Modulating the refractive index of the sapphire fiber with femtosecond laser technology is also a promising single-mode method [24,25]. Q. Guo reported helical SFBGs, while Y. Yu and A. Wang investigated the point-by-point inscription technique for parallel-integrated SFBGs [26,27,28]. Julian A. J. Fells has demonstrated a single-mode SFBG temperature sensor [29,30,31]. However, the current fabricated sapphire fiber Bragg gratings with better single-mode capability have relatively short grating lengths, which are not suitable for practical applications. Thomas E. Blue developed a novel internal cladding technique for sapphire fibers based on the Li^6^(n,α)H^3^ nuclear reaction [32]. However, this method is not feasible for mass production, as it not only requires a nuclear reactor environment but also incurs prohibitively high manufacturing costs. Y. Yu fabricated sapphire-derived fibers by the melt-in-tube method [33]. Because of the cladding material’s limitations, the fabricated sapphire-derived fiber grating was not applicable for use in extreme high-temperature environments of 1100 °C and above.

Curved sapphire fibers have been considered as a simple and low-cost technique for achieving single-mode capability of sapphire fibers. C. Zhan implemented a curved structure in ultra-thin (60 μm diameter) SFBGs, achieving higher-order mode suppression [34]. At a curvature radius of 10 mm, the SFBG achieved a resonant peak bandwidth below 2 nm, significantly improving the grating spectral characteristics while maintaining a thermal sensitivity of 12.4 pm/°C for temperature measurement. Y. Wang conducted curving experiments on large-core (150 μm diameter) SFBGs [35]. Their results showed that, at a 70 mm radius of curvature, the reflection spectrum exhibited a 3 nm bandwidth with negligible fundamental mode (FM) loss. This spectral bandwidth approaches that of conventional SMF-FBGs. Y. Yu revealed that curving helical SFBGs induces a mode-filtering effect and reduces spectral bandwidth [26]. Although the single-mode capability of the curved SFBG has been verified through experiments, there is a lack of theoretical and simulation analyses regarding the mode transmission of curved sapphire fibers.

In this paper, based on stress–optical effects and the conformal mapping technique, the equivalent refractive index theoretical model of a curved sapphire fiber is proposed and constructed. And the optimal parameters of a curved sapphire fiber finite element model are determined through the convergence analysis of the PML layer thickness and core mesh in COMSOL Multiphysics v.6.2. The variations in the mode electric field and the curved loss of different modes in curved sapphire fibers with a radius of curvature ranging from 0.01 m to 0.1 m are simulated. When the radius of curvature is 0.02 m, a five orders of magnitude difference between the curved loss of higher-order modes (HOMs) and that of the fundamental mode (FM) demonstrates optimal single-mode capability. In addition, a curved sapphire fiber transmission optical spectrum and a reflection optical spectrum test system for curved sapphire fiber Bragg grating (CSFBG) test systems are established. The single-mode capability of the curved sapphire fiber is characterized by comparing both the flatness of the transmission spectra and the mean squared error of spectral difference (MSESD) with that of single-mode fibers (SMFs). The experimentally measured spectral flatness and MSESD of curved sapphire fibers are minimized at a curvature radius of 0.02 m and are 3.22 dB and 0.042, respectively. When the radius of curvature is 0.02 m, the SNR (with the value of 6.11 dB) of CSFBGs is higher than those at 0.01 m and 0.03 m, and the 3 dB bandwidth reaches its minimum (with the value of 3.712 nm). In addition, a long-term stability test is conducted on the sapphire fiber and grating after exposure to 1600 °C. Under a constant curvature radius of 0.02 m for 120 h, neither component fractures nor shows significant spectral fluctuations, demonstrating their ultra-high long-term stability. Therefore, high-temperature tests from room temperature to 1600 °C are conducted, demonstrating the excellent high-temperature suitability of the CSFBG. This research provides crucial theoretical support for parameter selection and performance prediction of single-mode curved sapphire fibers and also offers guidance for the application of curved sapphire fibers in high-temperature in situ sensing.

## 2. Theoretical Model

**Figure 1:**
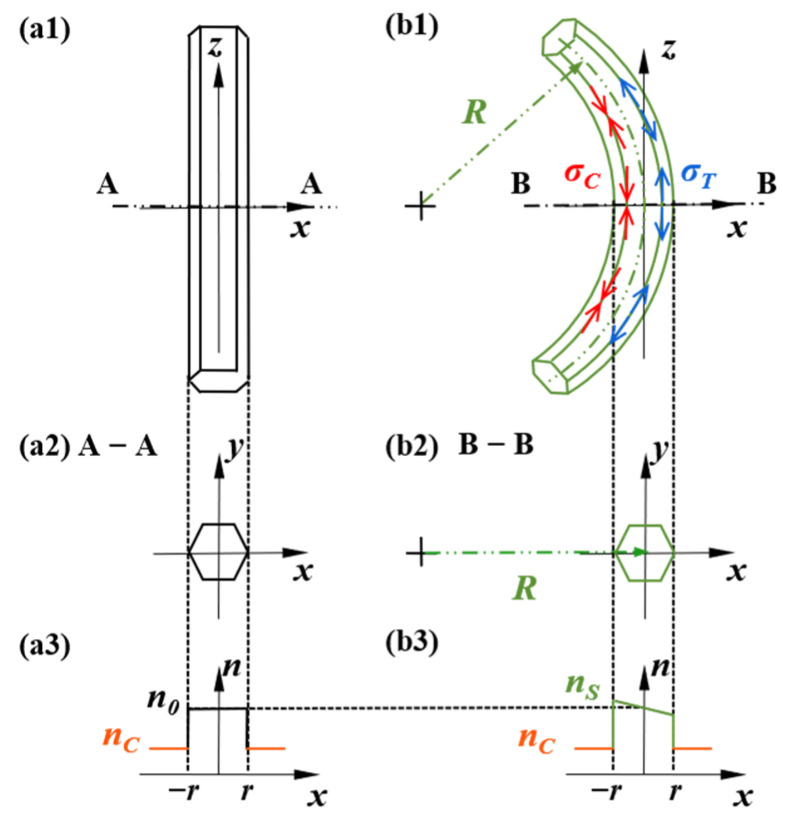
Sapphire fiber: (**a1**) axial top view; (**a2**) axial profile view; (**a3**) cross-section refractive index profile. Curved sapphire fiber: (**b1**) axial view stress distribution diagram; (**b2**) axial profile view; (**b3**) cross-section refractive index profile.

When the sapphire fiber is curved, the original refractive index of the sapphire fiber will change due to the stress–optical effect. Figure 1 illustrates the refractive index profiles along the cross-section of the sapphire fiber before and after curving. The sapphire fiber is a typical multimode step refractive index fiber, where r is the sapphire fiber radius; $n_{0}$ is the original refractive index of the sapphire fiber; and $n_{C}$ is the refractive index of the air cladding in Figure 1(a3). When curving stress is applied, both the inner and outer regions of the curved sapphire fiber are subjected to compressive stress $\delta_{C}$ and tensile stress $\delta_{T}$ respectively, as shown in Figure 1(b1).

The change in refractive index caused by stress–optical effects is

(1) $$△n_{S}(x)=-\frac{n_{0}^{3}x}{2R}\left[P_{12}-\upsilon (P_{11}+P_{12})\right]$$

where x is the position coordinate value of sapphire fiber cross-sectional, $n_{0}$ is original refractive index of sapphire fiber, R is radius of curvature, $P_{\mathrm{ij}}$ are components of the photo elastic tensor, $P_{11}$ = −0.237, $P_{12}$ = −0.027, υ is Poisson’s ratio.

The refractive index distribution of the cross-section of the curved sapphire fiber under an arbitrary radius of curvature can be analyzed and calculated by

(2) $$n_{S}(x)=n_{0}\left[1-\frac{n_{0}^{2}x}{2R}\left[P_{12}-\upsilon (P_{11}+P_{12})\right]\right]$$

As shown in Figure 1(b3), under the influence of curving stress, the refractive index of the curved sapphire fiber changes to $n_{S}(x)$.

**Figure 2:**
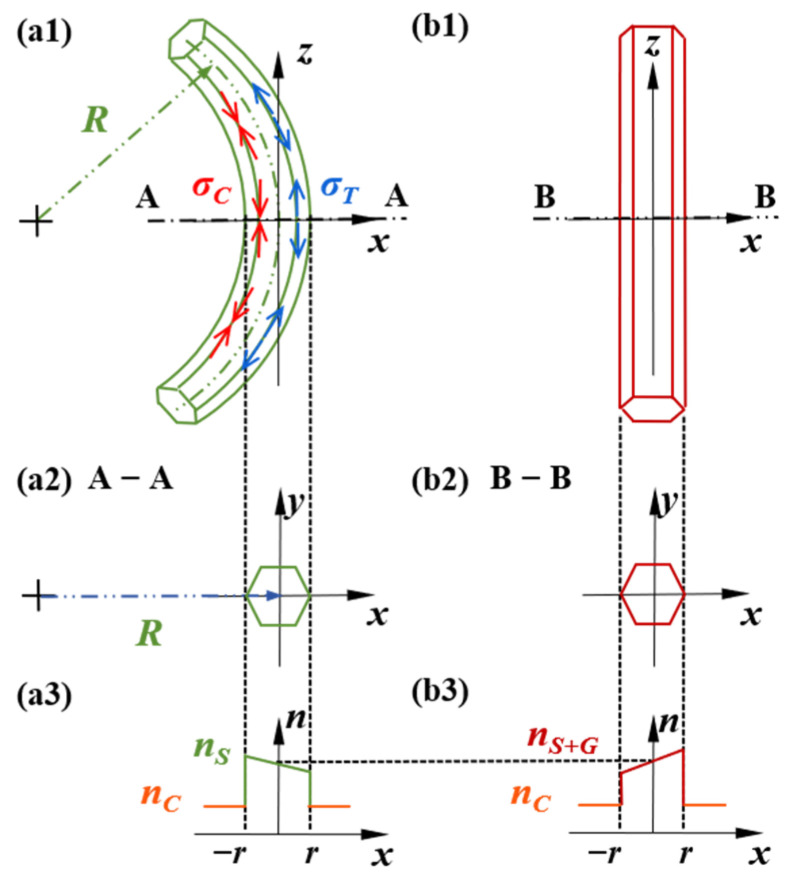
Curved sapphire fiber: (**a1**) axial view stress distribution diagram; (**a2**) axial profile view; (**a3**) cross-section refractive index profile. Equivalent sapphire fiber: (**b1**) axial top view; (**b2**) axial profile view; (**b3**) cross-section refractive index profile of the curved sapphire fiber.

In addition, changes in the optical path of the curved sapphire fiber can also affect the refractive index of the fiber. The refractive index of curved sapphire fibers can be equivalently mapped to that of straight fibers through the conformal mapping technique. This process is shown schematically in Figure 2.

For the radius of curvature R, the equivalent refractive index $n_{S+G}(x)$ of curved sapphire fiber is

(3) $$n_{S+G}\left(x\right)=n_{S}\left(x\right)\exp\left(\frac{x}{R}\right)$$

Since the radius of curvature R value measured in the experiment is much larger than the radius of the sapphire fiber (x≪R), the Taylor expansion of Equation (3) at $\frac{x}{R}\to 0$ can be simplified to

(4) $$n_{S+G}\left(x\right)=n_{S}\left(1+\frac{x}{R}+\frac{1}{2!}\frac{x^{2}}{R^{2}}+\frac{1}{3!}\frac{x^{3}}{R^{3}}+\ldots \right)\approx n_{S}\left(1+\frac{x}{R}\right)$$

To sum up, after considering both the stress–optical effects and the conformal mapping technique simultaneously, the equivalent refractive index theoretical model of the curved sapphire fiber can be obtained as

(5) $$n_{S+G}(x)=n_{0}\left[1-\frac{n_{0}^{2}x}{2R}\left[P_{12}-\upsilon (P_{11}+P_{12})\right]\right]\left(1+\frac{x}{R}\right)$$

The equivalent refractive index distribution of the curved sapphire fiber with a radius of curvature R is shown in Figure 2. When R is constant, the equivalent refractive index $n_{S+G}$ monotonically increases with the value of x. Since the sapphire fiber is usually in direct contact with air, the infinite air is regarded as the cladding material of the sapphire fiber. Therefore, $n_{c}$ will not change due to the curving structure of the sapphire fiber.

## 3. Simulation Analysis

In the simulation, the sapphire fiber core’s refractive index is defined using Equation (5), which implements the equivalent refractive index model for curved sapphire fibers. The parameters in the equation are assigned based on the material characteristics of the sapphire fiber to accurately capture the curving-induced refractive index variation. By directly varying the radii of curvature value within the model from 0.01 m to 0.1 m, the curved losses of different modes are simulated using the finite element method (FEM).

The single-crystal sapphire fiber consists of α-Al_2_O_3_ with a hexagonal lattice structure, yielding a hexagonal cross-section in the grown fiber. Accordingly, a two-dimensional hexagonal cross-sectional model of the curved sapphire fiber is constructed, with a core radius of 30 μm and a cladding radius of 90 μm. The incident light wavelength λ is set to 1550 nm; the original refractive index of the sapphire fiber ($n_{0}$) is set to 1.746; and the refractive index of the air cladding ($n_{C}$) is set to 1.000.

In practical applications, sapphire fiber utilizes the infinitely extending air as cladding. However, in simulation environments, the environment outside the fiber core is limited, which will cause deviations between the simulation results and the actual condition. To eliminate this discrepancy without altering the fundamental fiber model, a PML can be implemented to compensate for boundary effects and reduce simulation errors. As a non-reflective absorption layer, the PML ensures that incident light beams are not immediately reflected back but gradually attenuate until they are eventually completely absorbed.

In this simulation, the built-in PML of COMSOL is employed, where the thickness is a critical parameter. The convergence study of the PML thickness is conducted to calculate the influence of the PML thickness on the curved loss of the HOMs in the curved sapphire fiber. The thickness is set to kλ (k = 1, 2, …, 30), where k is a positive integer, and λ denotes the wavelength. Within the finite element analysis framework of this paper, the curved losses for sapphire fiber modes are derived from the imaginary part of its corresponding propagation constant Im(β), which is obtained by solving the generalized eigenvalue problem derived from Maxwell’s equations. The losses are calculated using the formula:

(6) Loss(dB/m)=20ln(10)Im(β)

**Figure 3:**
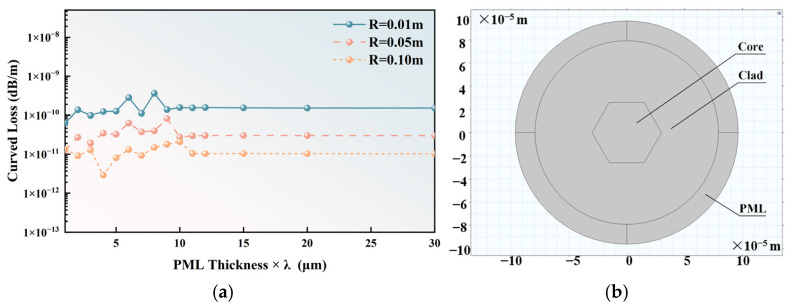
(**a**) Variation in the curved loss of HOMs in curved sapphire fiber versus the PML thickness. (**b**) Two-dimensional hexagonal cross-sectional model of curved sapphire fiber.

As shown in Figure 3a, the curved loss of HOMs fluctuates with increasing PML thickness when the latter is less than 11λ. When the PML thickness over 11λ, the relative error of the curved losses at different radii of curvature is less than 2%, and the curved losses tend to converge. However, excessive PML thickness increases computational demand, reducing simulation efficiency. Therefore, the optimal PML thickness is set to 11λ in the final model. The constructed two-dimensional hexagonal cross-sectional model of the curved sapphire fiber is presented in Figure 3b.

**Figure 4:**
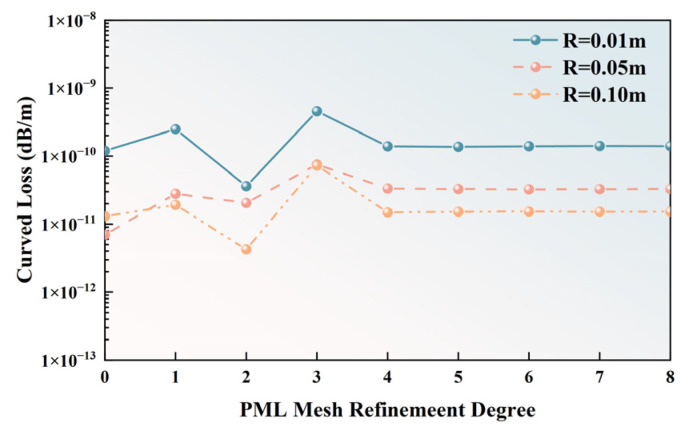
Variation in the curved loss of HOMs in curved sapphire fiber versus the refinement degree of PML mesh.

Appropriate meshing directly affects the accuracy and efficiency of simulation calculation. In the two-dimensional hexagonal cross-sectional model of the curved sapphire fiber, mapped meshing is applied to the PML region. As shown in Table 1, the details of the PML mesh refinement study include: mesh refinement degree (from 0 to 8), mesh setting method, mesh size parameter ranges (from max to min values), and degrees of freedom (DOF). The mesh refinement results are shown in Figure 4, which demonstrate that the HOM curved losses converge when the mesh is set to “extremely fine”, exhibiting a relative error of change of less than 2%.

**Figure 5:**
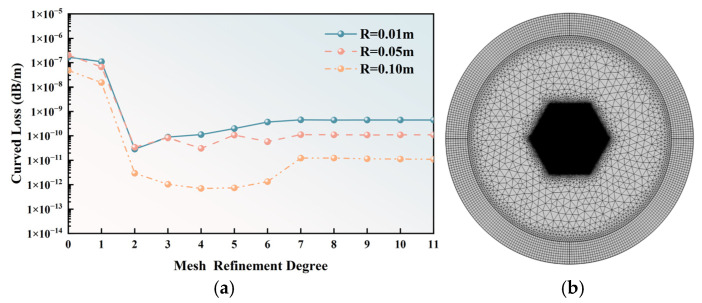
(**a**) Variation in the curved loss of HOMs in curved sapphire fiber versus the refinement degree of core mesh. (**b**) Mesh configuration of the 6 refinement degree.

Due to the multimode propagation and total internal reflection characteristics of the sapphire fiber, it is necessary to conduct detailed and differentiated processing of the core and cladding mesh in order to accurately calculate the curved loss of HOMs. Thus, a mesh refinement study was conducted specifically for the core region. The corresponding mesh settings are summarized in Table 2. Figure 5a plots the variation in the HOMs’ curved loss with mesh refinement degree (0–11). When the mesh size is refined to λ/15, the curved loss converges and stabilizes consistently across varying radii of curvature, exhibiting relative error below 2%. Consequently, the mesh configuration depicted in Figure 5b is adopted for subsequent simulations.

**Figure 6:**
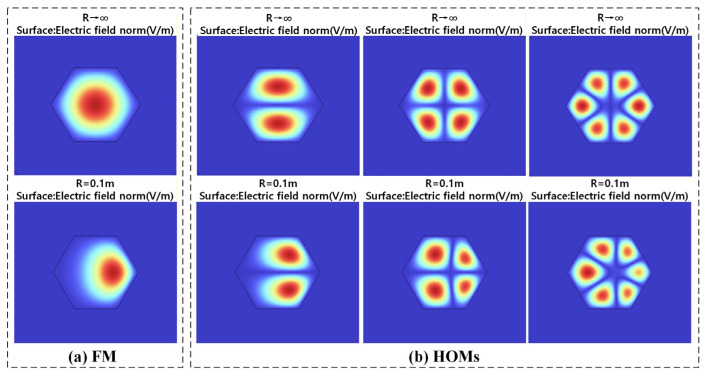
*R* = 0.1 m, comparison diagram of the electric field intensity centers of sapphire fiber before and after curving. (**a**) FM. (**b**) HOMs.

Under the λ/15 scale mesh sizing configuration, the electric field distribution of the curved sapphire fiber at a curvature radius of 0.1 m is compared with that of the straight sapphire fiber (R → ∞) in Figure 6. Obviously, the electric field distributions of both the FM and the HOMs of the curved sapphire fiber show a significant offset relative to the curve’s center of curvature. For curved sapphire fiber modes of this simulation, the center of curvature is located to the left of each figure. It is indicated that the curved structure of the sapphire fiber will cause mode curved loss.

**Figure 7:**
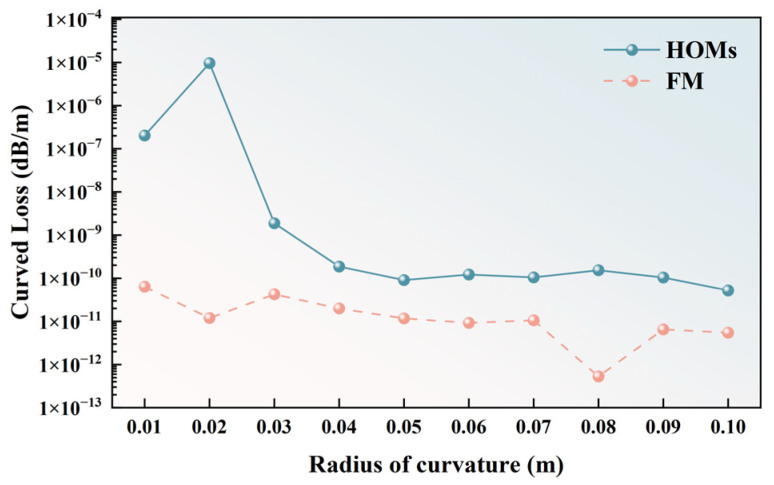
The curved loss diagram of FM and HOMs in curved sapphire fiber under different radii of curvature.

Furthermore, simulations were conducted on the mode curved loss of the curved sapphire fiber under different radii of curvature ranging from 0.01 m to 0.1 m. By substituting the propagation constants Im(β) of the HOMs and the FM into Equation (6), their respective curved loss values can be calculated. The results are shown in Figure 7. Under the same radius of curvature, the curved loss of the HOMs is always higher than that of the FM. Within the radius of curvature ranging from 0.1 m to 0.02 m, as the radius of curvature decreases, the curved loss of the HOMs gradually increases. When radius of curvature is less than 0.02 m, the HOMs’ curved loss begins to decrease again. That is to say, when the radius of curvature is 0.02 m, the HOMs’ curved loss reaches the maximum, and the FM loss is relatively small, with a difference spanning five orders of magnitude. This fully demonstrates that, at the curvature radius of 0.02 m, the single-mode capability of the curved sapphire fiber is optimal.

## 4. Experimental Investigation

**Figure 8:**
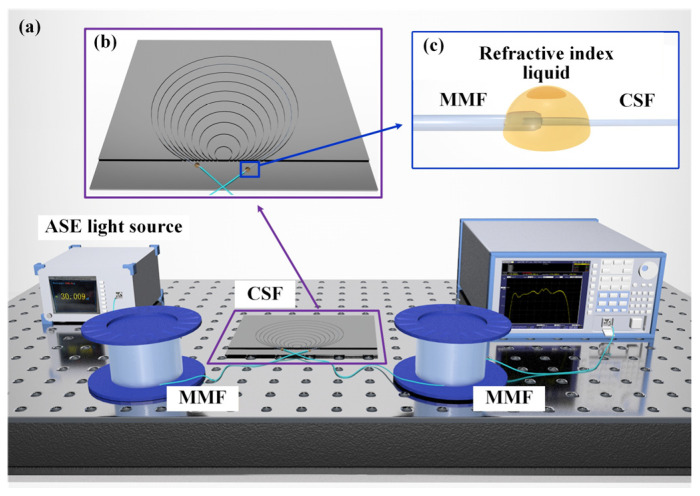
(**a**) Schematic diagram of the curved sapphire fiber curved loss experimental setup (ASE light source: amplified spontaneous emission light source, MMF: multimode fiber, OSA: optical spectrum analyzer). (**b**) Schematic diagram of the fusion point (CSF: curved sapphire fiber). (**c**) Schematic diagram of the fusion point between MMF and CSF.

To verify the single-mode capability of a curved sapphire fiber under different radii of curvature, a transmission optical spectrum test system for the curved sapphire fiber is established, as shown in Figure 8a. This system consists of an amplified spontaneous emission light source (ASE light source, GOLIGHT, Shenzhen, China), multimode fiber (MMF, YOFC, OM1, Wuhan, China) with a length of 500 m, the curved sapphire fiber (SF60-100, Jingying Inc., Xuzhou, China) and an optical spectrum analyzer (OSA, YOKOGAWA, Tokyo, Japan). In order to uniformly excite the propagation modes within the sapphire fiber and achieve a balanced and stable mode distribution, the 500 m MMF is connected into the experimental system. Although the optical path loss introduced by the 500-m MMF connection is objectively present [14], since all experimental conditions are kept consistent, it does not affect our relative evaluation of the single-mode effect caused by the curved structure. The curved sapphire fiber is achieved by fixing the sapphire fiber in a mold with grooves of different radii of curvature, as shown in Figure 8b. To minimize additional splicing loss at the fusion point between the curved sapphire fiber and the MMF, the fusion point is fully immersed in a refractive index liquid (schematically shown in Figure 8c), which can reduce the fiber splicing loss by 1.03 dB and significantly improve the optical coupling efficiency at the interface.

**Figure 9:**
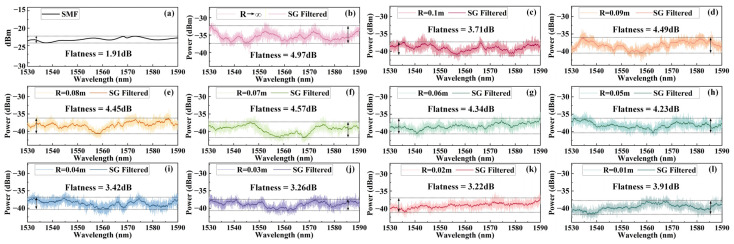
Transmission optical spectra of (**a**) SMF (single-mode fiber) and (**b**–**l**) curved sapphire fiber with different radii of curvature *R*: (**b**) *R* → ∞, (straight sapphire fiber); (**c**) *R* = 0.1 m; (**d**) *R* = 0.09 m; (**e**) *R* = 0.08 m; (**f**) *R* = 0.07 m; (**g**) *R* = 0.06 m; (**h**) *R* = 0.05 m; (**i**) *R* = 0.04 m; (**j**) *R* = 0.03 m; (**k**) *R* = 0.02 m; (**l**) *R* = 0.01 m.

The transmission optical spectra of the SMF and of the same curved sapphire fiber (total length of 50 cm, with the curved portion corresponding to an arc length of 25 cm) are measured for varying radii of curvature from 0.01 to 0.1 m, respectively. The results are shown in Figure 9. Due to the multimode characteristics of the sapphire fiber, many burrs appear in transmission optical spectrum. To reduce the calculation errors caused by the burrs, the Savitzky–Golay (SG) filtering method with a window size is carried out on the transmission optical spectrum of the curved sapphire fiber. In the transmission optical spectrum measurement of the curved sapphire fiber, the proposed method outperforms the neighborhood averaging method without causing significant distortion. As shown in Figure 9a, the SMF transmission optical spectrum has a spectral flatness of 1.91 dB. The spectral flatness of the curved sapphire fiber can be calculated based on the filtered transmission spectrum of the curved sapphire fiber. When the radius of curvature R is 0.02 m, the spectral flatness is the smallest (3.22 dB) and closest to the spectral flatness of the SMF.

**Figure 10:**
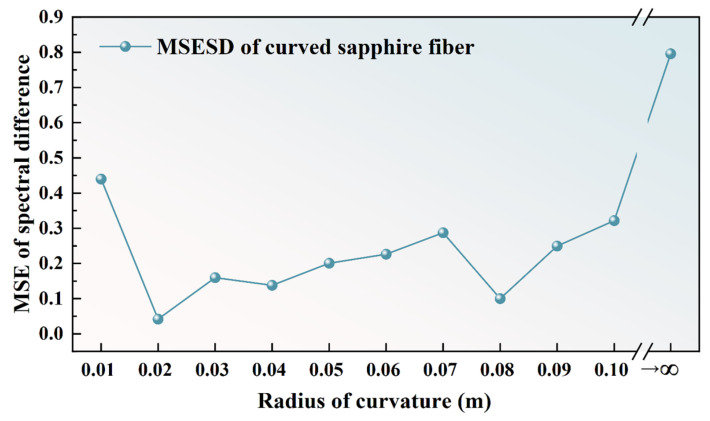
The variation graph of the MSESD compared with single-mode fiber spectra with different radii of curvature.

To further accurately characterize the spectral similarity between the curved sapphire fiber and the SMF transmission spectrum, a mean squared error of spectral difference (MSESD) method is proposed. This approach involves calculating the spectral difference between the SMF and the curved sapphire fiber (after SG filtered) at each wavelength sampling point. Then the mean squared error of these differences is calculated to quantify spectral deviation. As shown in Figure 10, when the radius of curvature is 0.02 m, the MSESD reaches the minimum, which is 0.042.

**Figure 11:**
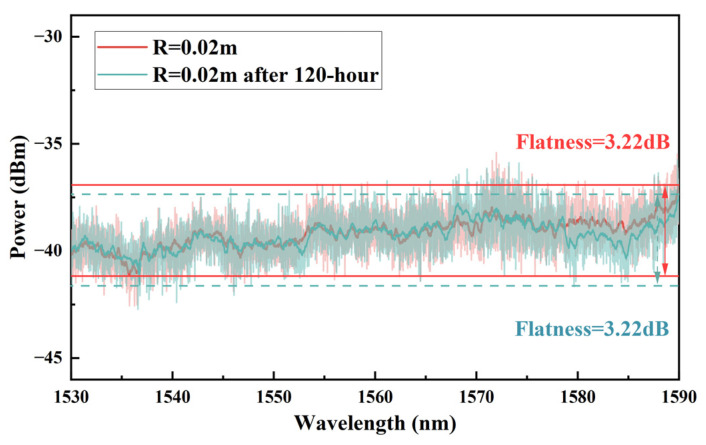
The transmission spectrum profile of the sapphire fiber before and after 120-h of curving test.

Based on pure curving theory derivation, the purely theoretical calculation value of the maximum curving stress at the outer edge of the sapphire fiber reaches 85 MPa under the optimal experimental curvature radius of 0.02 m, which is far lower than the minimum tensile strength limit of the sapphire fiber (2200 MPa) and will not cause mechanical damage to the fiber. A long-term stability test was performed on the sapphire fiber with a curvature radius of 0.02 m for 120 h. After the test, the fiber remained intact without fracture, demonstrating good structural stability. As shown in Figure 11, the transmission spectrum profile of the sapphire fiber was basically consistent before and after 120 h of curving test, verifying its excellent optical stability under long-term curving.

**Figure 12:**
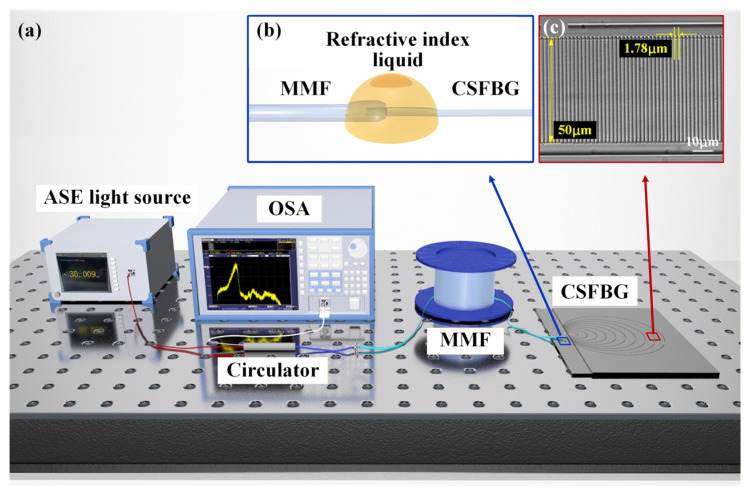
(**a**) Schematic diagram of the CSFBG reflection optical spectrum experimental setup. (**b**) Schematic diagram of the fusion point (CSFBG: curved sapphire fiber Bragg grating). (**c**) Microscopic image of a section of the grating region of the CSFBG.

Moreover, the single-mode capability of the curved sapphire fiber is characterized by the reflection optical spectrum of the curved sapphire fiber Bragg grating (CSFBG). A reflection optical spectrum test system for the CSFBG was established, as shown in Figure 12. This system consists of an ASE light source, an MMF with a length of 500 m, a circulator, the CSFBG and an OSA. In this system, the length of the curved sapphire fiber is 50 cm (with the curved portion corresponding to an arc length of 25 cm); the grating length of the SFBG used is 5000 μm; the period is 1.78 μm; and the line length is 50 μm. The grating region of the sapphire fiber Bragg grating (SFBG) is precisely positioned within the curving region.

**Figure 13:**
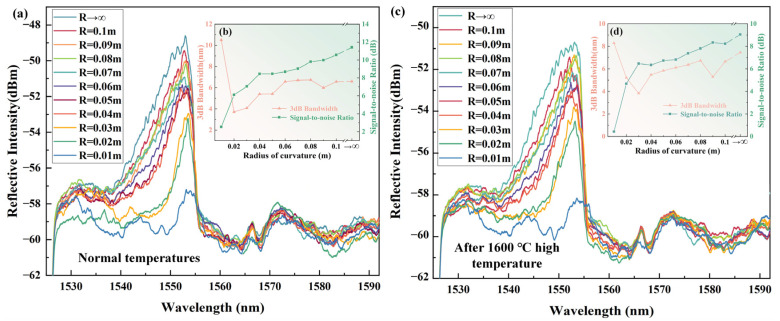
Normal temperatures: (**a**) reflection spectrum of CSFBG with different radii of curvature; (**b**) variation graph of the 3 dB bandwidth and SNR of CFBG with different radii of curvature. After 1600 °C high temperature: (**c**) reflection spectrum of CFBG with different radii of curvature; (**d**) variation graph of the 3 dB bandwidth and SNR of CSFBG with different radii of curvature.

Figure 13 shows the SFBG reflection spectrum at different radii of curvature, which is processed by SG filtering. The effective mode refractive index of the FM is larger than that of HOMs, so the wavelength of the FM is located on the longer-wavelength edge of the reflection peak, and the HOMs are located on the short-wavelength edge. Therefore, this phenomenon indicates that, as the radius of curvature decreases, the loss of the higher-order modes increases, while the loss of the FM is much smaller than that of the HOMs, which is consistent with the simulation analysis results. Additionally, as shown in Figure 13b, the signal-to-noise ratio (SNR) and the 3 dB bandwidth of the SFBG reflection spectrum also change with the reduction in the radius of curvature. The SNR of the SFBG’s reflection spectrum gradually decreases with the reduction in radius of curvature. This is due to the optical loss induced by fiber curving, which leads to a reduction in optical reflection intensity and a consequent decrease in the SNR. The curvature radius decreased from 0.1 m to 0.02 m, resulting in a reduction in the 3 dB bandwidth from 6.6 nm to 3.7 nm. This further verifies that the curved structure enhances the single-mode capability of the sapphire fiber.

The curving experimental results of the sapphire fiber and the SFBG are in good agreement with the simulation, demonstrating that the curved sapphire fiber exhibits optimal single-mode capability at the curvature radius of 0.02 m. Also the theoretical model of the curved sapphire fiber and its effectiveness in predicting high-order modes’ suppression characteristics are further confirmed.

To further verify the practical application potential of this technology, experimental investigations are carried out to evaluate its long-term curving stability and high-temperature adaptability.

Specifically, curving experiments on the SFBG after its 1600 °C high-temperature treatment were completed, with the results are shown in Figure 13c and d, respectively. As shown in Figure 13d, after high-temperature treatment at 1600 °C, the SFBG still exhibits a single-mode effect consistent with that at room temperature under different curvature radii, achieving optimal single-mode capability at a curvature radius of 0.02 m. Comparing Figure 13b,d, the signal-to-noise ratio (SNR) of the sapphire fiber grating decreases from 11.52 dB to 9.08 dB at a high temperature of 1600 °C compared with that at room temperature. This is because, when fabricating the SFBG by femtosecond laser direct writing, refractive index modulation is induced in the sapphire fiber, while residual stress also remains in the fiber. Under the high-temperature environment, the residual internal stress is released and redistributed, resulting in a reduction in the amplitude of the original refractive index modulation. In addition, the slight interaction between the ambient gas and sapphire at high temperatures causes a change in the fiber’s surface state, which increases scattering loss and thus leads to a decrease in SNR.

**Figure 14:**
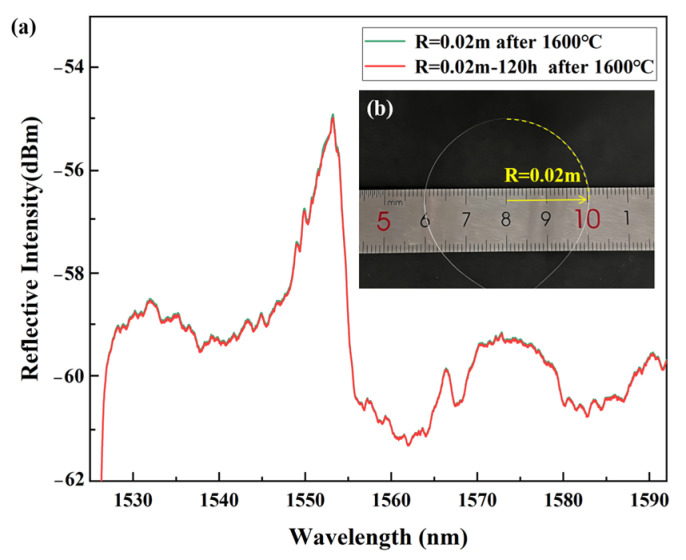
(**a**) Reflection spectra of SFBG before and after curving maintained for 120 h with a curvature radius of 0.02 m after 1600 °C high-temperature treatment. (**b**) Morphology of SFBG after being maintained for 120 h with a curvature radius of 0.02 m after 1600 °C high-temperature treatment.

In addition, the SFBG treated at 1600 °C underwent a 120 h long-term stability test. As shown in Figure 14a, after being maintained at a curvature radius of 0.02 m for 120 h, the reflection spectrum profile, central wavelength and reflectivity of the grating exhibit no obvious attenuation, and the spectral shape remains basically unchanged, demonstrating excellent long-term stability of its optical performance. As presented in Figure 14b, no fracture occurs in the SFBG after 120 h of continuous curving.

**Figure 15:**
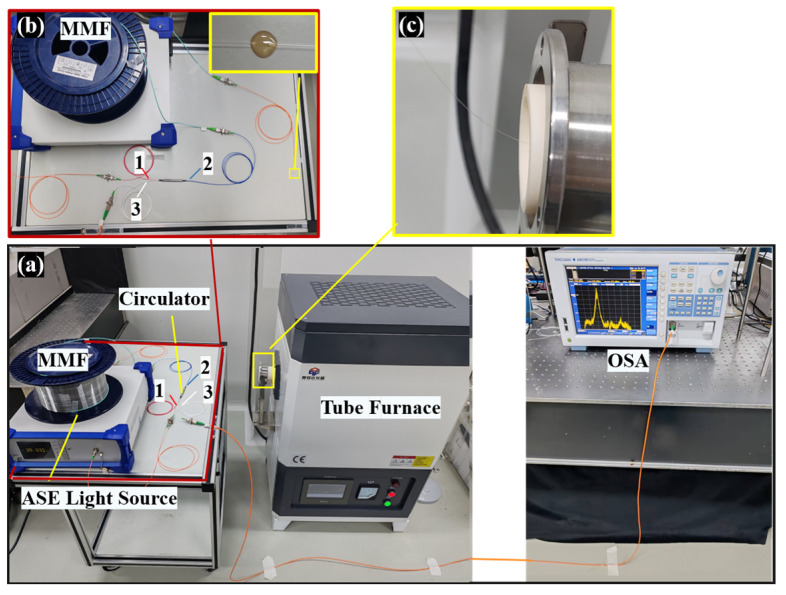
(**a**) High-temperature testing system diagram. (**b**) Photograph of the experimental platform (the thumbnail is an enlarged view of the splicing point). (**c**) Enlarged view of the furnace tube port.

As shown in Figure 15, the temperature response of a CSFBG (with a curvature radius of 0.02 m) was characterized by placing it in a tube furnace (AOFEIDA, SFDG-3-18, Zhengzhou, China). The SFBG is fixed by spot-bonding it with high-temperature inorganic adhesive into a grooved corundum mold with a curvature radius of 0.02 m. Light emitted from the ASE light source enters the circulator through Port 1, exits via Port 2, and propagates to the CSFBG. The light reflected by the CSFBG returns to the circulator through Port 2, is then directed to Port 3, and is finally detected by the OSA. Since the OSA is mounted on a fixed optical table, Port 3 of the circulator is connected to the OSA via a multimode patch cord to measure the reflection spectrum. The furnace temperature is varied between 22 °C and 1600 °C and held constant at each measurement point for 20 min.

**Figure 16:**
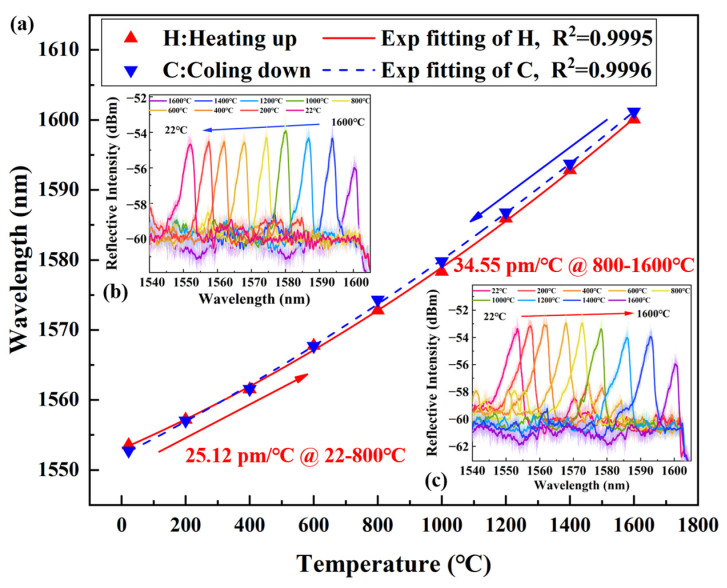
(**a**) The quadratic fitting curve of Bragg wavelength and temperature during the temperature cycling test from room temperature to 1600 °C. The variation in the reflection spectra with temperature during: (**b**) cooling process; (**c**) heating process.

Figure 16b,c show the reflection spectra of the CSFBG at different temperatures during the heating and cooling processes. Throughout the experiment, the reflection peaks consistently maintain a stable Gaussian distribution with a narrow bandwidth. Peak tracking is performed on the wavelength shift of the CSFBG during the heating and cooling processes, and the temperature dependence of the center wavelength shown in Figure 16a is obtained. The variation in the center wavelength with temperature is generally determined jointly by the thermo-optic coefficient and the thermal expansion coefficient. Over a wide temperature range, both coefficients vary with temperature, which leads to a nonlinear relationship between the center wavelength and temperature. Therefore, a quadratic fitting method is employed to describe the nonlinear characteristics of the center wavelength of the curved sapphire fiber Bragg grating as a function of temperature. The coefficients of determination (R^2^) of the quadratic fitting curves for the heating and cooling processes are 0.995 and 0.996, respectively. To characterize the temperature sensitivity of the CSBFG, a piecewise linear fitting method is used to obtain the average temperature sensitivity. The temperature sensitivity of the CSFBG is obtained in different ranges: 25.12 pm/°C from 22 to 800 °C and 34.55 pm/°C from 800 to 1600 °C.

**Figure 17:**
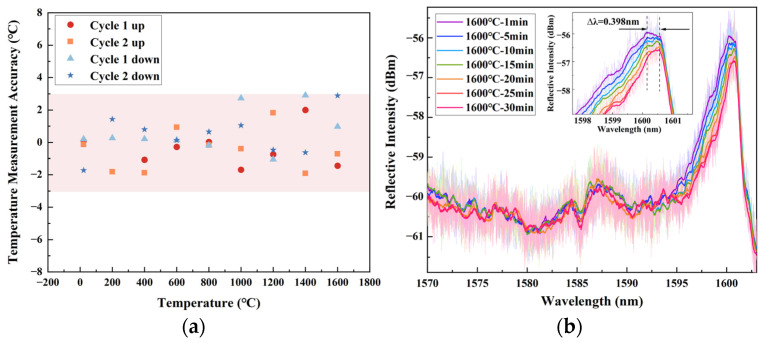
(**a**) The temperature deviation at each temperature point during two temperature cycles. (**b**) Reflection spectra of the CSFBG maintained at 1600 °C for 30 min. The illustration is a partial, enlarged view.

Using the quadratic fit obtained from Figure 16, we used a quadratic fit *λ_B_ =* A *+* B*T +* C*T^2^*, with A, B, and C found to be 1553.01 nm, 19.99 pm/°C, and 5.96 × 10^−6^ pm/°C^2^ respectively. The sensitivity varies between 21.31 and 37.88 pm/°C. Also shown in Figure 17a is the temperature deviation at each temperature point, obtained by subtracting the Bragg wavelength obtained from a quadratic fit and then dividing by the slope of the characteristic curve at that point. The variation is within ±2.98 °C.

We conducted a long-term stability test. A sapphire fiber Bragg grating with a curvature radius of 0.02 m is placed in a tube furnace for an extended experiment lasting 30 min at 1600 °C. Its spectral response is recorded at regular intervals over the 30 min period to monitor the drift of the resonance wavelength in the reflection spectrum. The test results, as shown in Figure 17b, indicate that the reflection spectrum of the sensor remains stable throughout the 30 min of continuous testing. Based on the initial Bragg wavelength position, the maximum drift in the Bragg wavelength over the 30 min period is 0.398 nm, demonstrating good stability.

In summary, as shown in Table 3, the technologies for achieving single-mode capability in sapphire fibers can be divided into the following categories: core diameter reduction method, refractive index modulation method, cladding fabrication method, and curved fiber method. The core diameter reduction method is theoretically feasible, but its limited maturity in existing fabrication processes, low efficiency, and poor mechanical strength make it difficult to make it a mainstream practical solution. The refractive index modulation method fabricates single-mode structures inside the sapphire fiber through femtosecond laser direct writing. Although this method offers significant advantages in achieving single-mode operation, it is constrained by high equipment costs, difficult fabrication, and challenges in mass production. The cladding fabrication method imposes extremely stringent requirements on the environment and reaction conditions, which limits its widespread adoption; moreover, it is hindered by the limitations of cladding materials, making it difficult to achieve high-temperature sensing. In contrast, the curved fiber method provides a structurally simple, convenient, and economical alternative. However, previous studies on curved sapphire fibers have mostly focused on experimental testing, lacking systematic theoretical analysis. To address this, this paper innovatively proposes an equivalent refractive index theoretical model for the sapphire fiber and simulates the bending loss of different modes under various bending radii, thereby providing a theoretical basis for subsequent experimental research. On this basis, the curved sapphire fiber Bragg grating was subjected to high-temperature testing up to 1600 °C, and the results demonstrate that it exhibits good sensing performance.

## 5. Conclusions

This paper proposes and develops an equivalent refractive index theoretical model for curved sapphire fibers based on stress–optic effects and the conformal mapping technique. This theoretical model can calculate the equivalent refractive index distribution of a curved sapphire fiber cross-section under an arbitrary curvature radius. Based on this model, the curved loss of different modes of the curved sapphire fiber is calculated by FEM under different radii of curvature ranging from 0.01 to 0.1 m. The results demonstrate that, when the radius of curvature is 0.02 m, the HOMs exhibit the highest curved losses, with five orders of magnitude difference compared with that of the FM. In addition, at a 0.02 m radius of curvature, the measured spectral flatness of the curved sapphire fiber through the transmission optical spectrum test system reaches its minimum value (3.22 dB), showing close agreement with the spectral flatness of SMFs. And the lowest MSESD is 0.042, indicating the highest spectral consistency. Furthermore, the reflection spectrum measurements of the CSFBG experimentally confirm that the sapphire fiber exhibits optimal single-mode capability at a curvature radius of 0.02 m, where the 3 dB bandwidth of the CSFBG is as low as 3.7 nm. Meanwhile, the variation in the short-wavelength edge of the reflection spectrum with the curing radius demonstrates the effective suppression of higher-order modes in the sapphire fiber under curving conditions. Additionally, a 120 h long-term stability test was performed on the SFBG after 1600 °C treatment under a constant curvature radius of 0.02 m. The results show no fracture and no significant spectral fluctuation, confirming its exceptional long-term stability. The curved sapphire fiber Bragg grating demonstrates stable operation at temperatures as high as 1600 °C, achieving a measurement accuracy of ±2.98 °C. This study provides theoretical guidelines for assessing the single-mode capability of curved sapphire fibers. A curved sapphire fiber is a simple and effective method to achieve single-mode capability, which has great potential for in situ sensing in extreme high-temperature environments.

## Figures and Tables

**Table 1:** Setting details of PML mesh refinement degree.

Mesh Refinement Degree	Mesh Setting Method	Mesh Size Parameter Ranges	DOF
0—Normal	Physics controlled	12.9 μm–57.7 nm	523,589
1—Fine	Physics controlled	10.2 μm–57.7 nm	525,147
2—Finer	Physics controlled	7.12 μm–24 nm	531,691
3—Extra fine	Physics controlled	3.85 μm–14.4 nm	541,361
4—Extremely fine	Physics controlled	1.92 μm–3.85 nm	582,007
5—λ	User controlled	1.55 μm–3.85 nm	616,759
6—λ/5	User controlled	310 nm–3.85 nm	2,060,419
7—λ/15	User controlled	100 nm–3.85 nm	147,451,217
8—λ/20	User controlled	75 nm–3.85 nm	292,753,062

**Table 2:** Setting details of core mesh refinement degree.

Mesh Refinement Degree	Mesh Setting Method	Mesh Size Parameter Ranges	DOF
0—Normal	Physics controlled	8 μm–36 nm	4175
1—Fine	Physics controlled	6.36 μm–36 nm	58,071
2—Finer	Physics controlled	4.44 μm–15 nm	65,365
3—Extra fine	Physics controlled	2.4 μm–9 nm	79,785
4—Extremely fine	Physics controlled	1.92 μm–3.85 nm	82,409
5—λ	User controlled	1.55 μm–3.85 nm	84,195
6—λ/5	User controlled	310 nm–3.85 nm	529,703
7—λ/15	User controlled	100 nm–3.85 nm	4,706,841
8—λ/20	User controlled	75 nm–3.85 nm	8,428,055
9—λ/25	User controlled	62 nm–3.85 nm	12,324,847
10—λ/30	User controlled	50 nm–3.85 nm	18,961,753
11—λ/35	User controlled	45 nm–3.85 nm	24,025,475

**Table 3:** Performance comparison between the different single-mode capability technologies of sapphire fiber.

Methods	Authors	High-Temperature Suitability	Fabrication Difficulty and Cost	Temperature Sensing Performance Metric—Sensitivity	Ref.
Core diameter decreasing method	C. Hill et al.	≤1400 °C	High (long reaction time, high preparation difficulty)	26.5 pm/°C (@22-1600 °C)	[21]
	T. Wang et al.	d = 16µm: no test; d = 30µm: ≤1600 °C	High (extremely complex process, extremely high requirements for equipment precision)	d = 16µm: no test; d = 30µm: 40.85 pm/°C (@ 1600 °C)	[23]
Refractive index modulation	Q. Guo et al.	≤1600 °C	High (expensive femtosecond laser equipment, high preparation difficulty)	24.42 pm/°C (@ 0–500 °C) 29.26 pm/°C (@ 500–1000 °C), 35.55 pm/°C (@ 1000–1600 °C)	[26]
	M. Wang et al.	≤1200 °C	High (expensive femtosecond laser equipment, complex structural design)	35.1 pm/°C (@ 1600 °C)	[31]
Cladding fabricating method	T. E. Blue et al.	≤1500 °C	Extremely high (depends on nuclear reactors)	No test	[32]
	Y. Yu et al.	≤1000 °C	Moderate (mature tube melting process)	15.64 pm/°C (@ 26–1000 °C)	[33]
Curved fiber method	C. Zhan et al.	≤1600 °C	Low (easy to implement)	No test	[34]
	Y. Wang et al.	No test	Low (easy to implement)	No test	[35]
	F. He et al.	≤1600 °C	Low (easy to implement, the optimal curvature radius can be predicted through simulation)	25.12 pm/°C (@ 22–800 °C) 34.55 pm/°C (@ 800–1600 °C) 37.88 pm/°C (@ 1600 °C)	This work

## Data Availability

Dataset available on request from the authors.
